# Multidrug resistance to commonly prescribed antibiotics in *Escherichia coli* isolated from barbecued beef (*Suya*) sold in a Nigerian City

**DOI:** 10.11604/pamj.2021.39.50.25502

**Published:** 2021-05-19

**Authors:** Danladi Walong Datok, David Ishaleku, Paul Alumbugu Tsaku, Elisha Obakas Agya, Moses Peter Adoga

**Affiliations:** 1 Department of Microbiology, Nasarawa State University, Keffi, Nigeria,; 2 Standards Organisation of Nigeria (SON), Federal Capital Territory (FCT) Office, Wuse Zone 4, Abuja, Nigeria

**Keywords:** Drug resistance, *Escherichia coli*, hygiene, public health, *Suya*, Nigeria

## Abstract

**Introduction:**

Suya, a form of barbecued meat widely consumed in Nigeria is a rising source of concern for the dissemination of pathogens and antibiotic resistance.

**Methods:**

this study was carried out to determine the antibiotic resistance profile of Escherichia coli (E. coli) isolated from Suya sold in Karu Local Government Area, Nasarawa State, Nigeria. A total of three hundred (300) Suya samples were collected and screened for the presence of E. coli. An antibiotic susceptibility study was carried out on the isolated bacteria to determine their resistance profiles.

**Results:**

the overall isolation and occurrence of E. coli was 13.3%. The isolated organisms were most resistant to Ampicillin (100%) followed by Amoxicillin/Clavulanic acid (95%), Ciprofloxacin (92.5%), Nitrofurantoin, Imipenem and Ceftriaxone (85%), Cefixime (80%), Streptomycin (77.5%), and Cotrimoxazole (77%), with a low level of resistance recorded against Gentamycin (5%). Most of the E. coli isolates had multiple resistance (MAR) to at least five antibiotics (MAR Index of = 0.5) and the most frequent MAR Index was 0.8 with 37.5% occurrence. The most frequently encountered resistance phenotype was Nitrofurantoin-Imipenem-Streptomycin-Ciprofloxacin-Ceftriaxone-Amoxicillin/clavulanic acid-Cefixime-Ampicillin. The E. coli isolates categorised based on drug resistance classes were Multi-Drug Resistance, MDR (97.5%), Pan Drug Resistance, PDR (2.5%), Non-Multi-Drug Resistance, NMDR (0.0%) and Extensive Drug Resistance, XDR (0.0%).

**Conclusion:**

these findings indicate a potential danger of multidrug resistant organisms in circulation. Antibiotics stewardship and drug resistance surveillance is strongly recommended for all stakeholders

## Introduction

*Suya* is a popular traditionally processed, ready-to-eat barbecued meat product, which is usually served or sold along the streets and served at hospitality industries such as social functions, club houses, picnics and restaurants. It is an ideal source of energy and animal protein. The practice of processing *Suya* is known from time immemorial [1] and it is consumed and perceived by consumers to be hygienic and uncontaminated. It is so named by the Hausa speaking natives of the northern Nigeria but its consumption transcends borders of ethnicity, tribe or religion across Nigeria, West Africa and beyond [2, 3].

Despite the wide distribution of this delicacy, little or no attention is paid to its safety, quality and hygiene; hence the possible occurrence of food borne disease [4, 5]. It is fairly recent that studies probing the microbiological and public health integrity of *Suya* began to emerge [2, 3, 6, 7].

The presence of *E. coli* in foods that are ready for consumption is indicative of poor hygiene and contamination could have arisen from human or animal faecal sources [2]. Most non-pathogenic but virulent strains of *E. coli* which causes several kinds of ailments, including food-borne infections have been identified [8]. *E. coli* strains that are widely resistant to commonly prescribed antibiotics have been reported [6, 9]. This study examined the antibiotic resistance profile of *E. coli* isolated from *Suya* sold in Karu Local Government Area of Nasarawa State, Nigeria.

## Methods

### Study Area

This study was carried out at Karu Local Government Area of Nasarawa State, Nigeria in May, 2020. It is located between latitudes 8° 5´ N and 10° 42´ N and longitudes 9° 25´E and 7°54´E of the Greenwich Meridian covering a spatial extent of about 2,640 km^2^ with a population of 205,477 [10]. It shares its western, northern, eastern and southern boundaries with Abuja - the Federal Capital Territory, Kaduna state, Keffi and Nasarawa local government areas of Nasarawa state respectively [11]. The major towns in Karu LGA are; Mararaba, One Man Village, Ado, New Nyanya, Nyanya Gwandara and Masaka.

### Sample collection

Three hundred (300) *Suya* samples were randomly purchased at Masaka, Ado, Mararaba, New Nyanya, Nyanya Gwandara and One Man Village (50 samples from each location). The samples were aseptically wrapped and labelled with date, time of sampling, area of sampling in a foil paper and transported to the Microbiology Laboratory of Nasarawa State University, Keffi, Nigeria for analysis.

### Isolation and identification of *Escherichia coli*

Primary culture was prepared by aseptically inoculating 1 g of the *Suya* sample in 10 ml of nutrient broth and incubated at 37°C for 24 hrs. To obtain pure cultures, samples from the primary culture were sub-cultured on Levine Eosin Methylene Blue (EMB) Agar (HiMedia, India) plates by streaking and incubated at 37°C. The plates were observed after 24 hrs incubation; greenish metallic sheen indicates the presence of *Escherichia spp* [12]. API 20E (BiomerieuxTM) kit was used for biochemical identification of *E. coli* following manufacturer´s instructions.

### Antibiotics susceptibility test

The antibiotics susceptibility test of the *E. coli* isolates was carried out using Kirby-Baeur disk diffusion method. The antibiotic disks were firmly placed on the sterile Mueller Hinton Agar (MHA) plates, seeded with test organisms standardized to 0.5 McFarland´s turbidity and incubated at 37 ^o^C for 24 hrs. Diameter of zones of inhibition was then measured to the nearest millimetre and reported in accordance with the antimicrobial susceptibility breakpoint of CLSI [13].

### Determination of Multiple Antibiotic Resistance (MAR) Index

The MAR Index was determined according to the method of Krumperman [14] and Paul *et al*. [15]. From the result of the antibiotic susceptibility test, MARI was calculated as:

### Data analysis

Data obtained was entered into Microsoft Excel^TM^ 2016 and subsequently SPSS v25 for statistical computation. The difference in the number of the bacterial isolates was compared from the different locations using p < 0.05 as the threshold for statistical significance.

## Results

**Figure 1:**
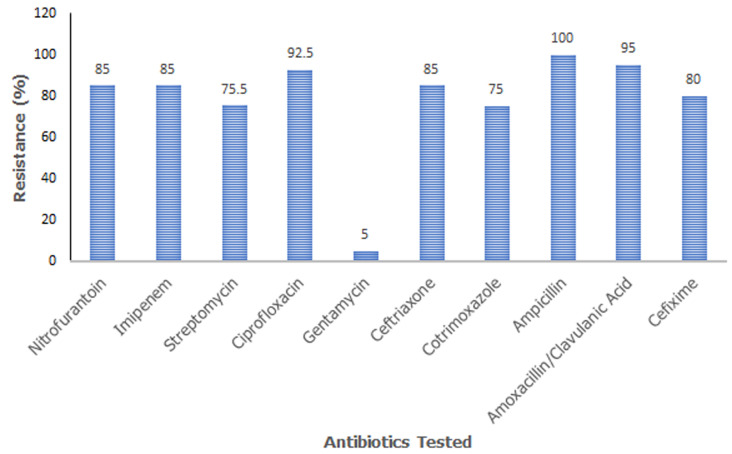
antibiotic resistance profile of *Escherichia coli* isolated from *Suya* sold in Karu Local Government Area of Nasarawa State, Nigeria

This study determined the presence of *E. coli* in *Suya* sold in Karu local government area of Nasarawa state, Nigeria. Out of 300 samples obtained for this study, *E. coli* was isolated from 40 out of the 300 samples which is equivalent to a 13.3% isolation rate. The isolation rate from major towns within the study area is outlined in Table 1. The antibiotic resistance profile in this study indicates that all 40 *E. coli* isolated from *Suya* within Karu, Nasarawa state was resistant to ampicillin. Out of the ten antibiotics tested (i.e. Nitrofurantoin, Imipenem, Streptomycin, Ciprofloxacin, Gentamycin, Ceftriaxone, Cotrimoxazole, Ampicillin, Amoxicillin/Clavulanic acid and Cefixime), resistance levels = 75% was observed against all but one of the antibiotics. Only 5% resistance was recorded against gentamycin (Figure 1).

**Table 1:** occurrence of *Escherichia coli* from *Suya* samples in relation to locations

Sample location	No sampled	No. (%) isolates	P-value
**A**	**52**	**8 (15.4)**	0.0013
**B**	**62**	**10 (16.1)**	
**C**	**40**	**5 (12.5)**	
**D**	**50**	**8 (16.0)**	
**E**	**50**	**4 (8.0)**	
**F**	**46**	**5 (10.9)**	
**Total**	**300**	**40 (13.3)**	

P-value less than 0.05 indicates statistical significance in the isolates from different location using X2 test. A-Masaka, B-Mararaba, C-Ado, D-New Nyanya, E-Nyanya Gwandara, F-One Man Village

The antibiotics resistance phenotypes and the multiple antibiotics resistance index (MARI) observed in this study are presented in Table 2. MARI = 0.5 was observed which implies that; all the isolated *E. coli* from *Suya* sold in Karu local government area of Nasarawa state, Nigeria are resistant to at least five out of the 10 antibiotics tested in this study. The resistance phenotypes with the highest rate of occurrence were F Ipm S Cip Cro Sxt Amc Cfm Amp 7(17.5%) and F Ipm S Cip Cro Amc Cfm Amp 7(17.5%). Thirty-nine (97.5%) of the *E. coli* isolates were of the multidrug resistance (MDR) class, while 1(2.5%) of the isolates was pan-drug resistant (PDR) (Table 3).

**Table 2:** antibiotic resistance phenotypes of *Escherichia coli* isolated from *Suya* sold in Karu Local Government Area of Nasarawa State, Nigeria

Antibiotic resistance phenotypes	No. (%) Isolates (n = 40)	MARI
F Ipm S Cip Cn Cro Sxt Amc Cfm Amp	1(2.5)	1.0
F Ipm S Cip Cro Sxt Amc Cfm Amp	7(17.5)	0.9
F Ipm S Cip Cro Amc Cfm Amp	7(17.5)	0.8
F S Cip Cro Sxt Amc Cfm Amp	1(2.5)	0.8
F S Cip Cro Sxt Amc Cfm Amp	1(2.5)	0.8
F Ipm Cip Cn Sxt Amc Cfm Amp	1(2.5)	0.8
F Imp S Cip Cro Sxt Amc Amp	2(5.0)	0.8
Ipm S Cip Cro Sxt Amc Cfm Amp	2(5.0)	0.8
Ipm S Cip Cro Sxt Cfm Amp	1(2.5)	0.7
F Ipm Cip Sxt Amc Cfm Amp	2(5.0)	0.7
F Ipm Cip Cro Sxt Amc Amp	1(2.5)	0.7
F Ipm Cip Cro Sxt Cfm Amp	1(2.5)	0.7
F Ipm S Sxt Amc Cfm Amp	1(2.5)	0.7
F Imp S Cip Amc Cfm Amp	1(2.5)	0.7
F S Cip Cro Amc Cfm Amp	2(5.0)	0.7
F S Cro Amc Cfm Amp	2(5.0)	0.6
F Cip Cro Sxt Amc Amp	1(2.5)	0.6
F Ipm Cip Cro Cfm Amp	1(2.5)	0.6
F Imp Cip Cro Sxt Amp	1(2.5)	0.6
Ipm S Cro Amc Cfm Amp	1(2.5)	0.6
Ipm S Cro Cfm Amp	1(2.5)	0.5
F S Cip Sxt Amp	1(2.5)	0.5
S Cip Cro Amc Amp	1(2.5)	0.5

F= Nitrofurantoin, Ipm= Imipenem, S= Streptomycin, Cip= Ciprofloxacin, Cn =Gentamycin, Cro= Ceftriaxone, Sxt = Cotrimoxazole, Amp= Ampicillin, Amc= Amoxicillin, Cfm= Cefixime, MARI= Multiple Antibiotics Resistance Index

**Table 3:** antibiotic resistance classes of *Escherichia coli* isolated from *Suya* sold in Karu Local Government Area of Nasarawa State, Nigeria

Classes of Antibiotics Resistance	No. (%) E. coli (n=40)
MDR	39 (97.5)
XDR	0(0.0)
PDR	1 (2.5)
NMDR	0(0.0)

NMDR = Non-Multi-Drug Resistance (non-susceptible to a class of antimicrobial categories); MDR = Multi-Drug Resistance (non-susceptible to ≥ 1 agent in ≥ 3 antimicrobial categories); XDR = Extensive Drug Resistance (non-susceptible to ≥ 1 agent in all but ≤ 2 antimicrobial categories); PDR = Pan Drug Resistance (non-susceptible to all antimicrobial listed) (Magiorakos et al., 2012)

## Discussion

In this study, 40 (13.3%) *E. coli* isolates were recovered from 300 *Suya* sampled at Karu Local Government Area of Nasarawa. The occurrence of *E. coli* from major towns of the local government area was Mararaba (16.1%) followed by New Nyanya (16.0%), Masaka (15.4%), Ado (12.5%), One Man Village (10.9%), and Nyanya Gwandara (8.0%). The isolation of *E. coli* from the *Suya* samples in this area suggests poor handling the meat, poor sanitation practices and lack of education among food handlers which if not controlled might constitute risk for food borne illness. Other possible factors could be due to the dusty nature of the area where meat shops are located or *Suya* displayed on tables with no wire mesh or net protecting it from flies.

This finding corroborates previous reports which found spiced, ready-to-eat meat products to be bacterial-laden [2, 3, 6, 7, 16, 17]. Magwira *et al*. [18] in Botswana reported that *E. coli* isolated from meat had occurrences of 2.3% whereas, 3.8% occurrence of *E. coli* was reported by of Abd El-Atty and Meshref [19] in Egypt, with a prevalence of 2.0% in sausage which was attributed to contamination from faeces of infected animals as well as unsatisfactory hygienic measures during handling. The lowest occurrence recorded at Nyanya Gwandara might be due to the lower population in that area as theorized by Ologhobo *et al*. [20], or it may be that most *Suya* vendors warm the product before serving their customers.

In this study, the *E. coli* had high rates of resistance to most of the tested antibiotics with the exception of Gentamycin which had only 5.0% resistance. The high antibiotic resistance observed could be that they are the most commonly available antibiotics used for treatment as well as growth promoters and in routine chemoprophylaxis among livestock as speculated by Olatoye [21]. This portends a major challenge in both human and animal medicine because these drugs are commonly used in the treatment of common human ailments.

All the isolates in this study were Multiple Antibiotic Resistant (MAR) with MAR Index of = 0.5 and the most frequent MAR Index was 0.8 with occurrence of 37.5%. Most of the *E. coli* isolates were Multi Drug Resistance (MDR) with the order of occurrence as: MDR (97.5%) > PDR (2.5%) >XDR (0.0%) and NMDR (0. 0%). This shows that the indiscriminate use of antibiotics in this environment may eventually supersede the drug sensitive microorganisms from an antibiotic saturated environment [22]. This is worrisome as Mishra *et al*. [23] asserted that MAR index of 0.4 and above is associated with human faecal source contamination and therefore sensitivity patterns and treatment should be guided by laboratory investigations.

## Conclusion

This study evaluated a total of 300 *Suya* samples and 40 *E. coli* were isolated and identified using standard microbiological techniques. The overall isolation and occurrence of *E. coli* was 13.3% with the highest from Mararaba (16.1%) while Nyanya Gwandara had the least with 8.0%. The isolates were most resistant to Ampicillin (100%) while Gentamycin had the least with 5% resistance. The multiple antibiotic resistance pattern of the isolates showed that most of the *E. coli* were resistant to more than five antibiotics, hence exhibited Multi-Drug Resistance (MDR) strains with the order of occurrence as: Multi-Drug Resistance, MDR (97.5%)? Pan Drug Resistance, PDR (2.5%)? Non-Multi-Drug Resistance, NMDR (0.0) and Extensive Drug Resistance, XDR (0.0). All the isolates showed Multiple Antibiotic Resistance (MAR) with MAR Index of = 0.5 and the most frequent MAR Index was 0.8 with occurrence of 37.5%. These findings indicate the possibility of the dissemination of multidrug resistant food-borne pathogens such as diarrhoeagenic *E. coli* within the studied locality. It is recommended that proactive measures should be taken by the government and civil society organizations to educate the meat vendors and the general populace on proper hygienic handling *Suya*. The public should also be educated on the proper usage of antibiotics in order to reduce the continuous distribution of multidrug resistance.

### What is known about this topic

- Escherichia coli is a regular foodborne pathogen;
- Antibiotics resistance by E. coli to frequently prescribed antibiotics is common phenomenon.

### What this study adds

- In this study, 13% occurrence of E. coli was found in barbecued beef (Suya ) which is ready for consumption in Karu area of Nasarawa state, central Nigeria;
- Ninety-seven-point five percent (97.5%) of the E. coli isolated from the Suya are multidrug resistant.
